# Interaction patterns among risk factors for bladder cancer in adults with type 2 diabetes managed in primary care: a retrospective cohort study

**DOI:** 10.3399/BJGPO.2025.0028

**Published:** 2026-02-11

**Authors:** Sarah Tsz Yui Yau, Eman Yee Man Leung, Chi Tim Hung, Martin Chi Sang Wong, Ka Chun Chong, Albert Lee, Eng Kiong Yeoh

**Affiliations:** 1 JC School of Public Health and Primary Care, The Chinese University of Hong Kong, Shatin, Hong Kong, China

**Keywords:** bladder cancer, diabetes mellitus, primary health care, interaction, public health, survival analysis

## Abstract

**Background:**

Previous studies have shown that patients with type 2 diabetes have a higher risk of developing bladder cancer than the general population. However, little is known about how different risk factors interact to influence the risk of bladder cancer among patients with diabetes.

**Aim:**

To explore the interaction patterns among factors associated with the risk of bladder cancer incidence among patients who received diabetes management in primary care.

**Design & setting:**

A retrospective cohort study was performed using territory-wide electronic health records of Hong Kong. Patients who received diabetes care in general outpatient clinics between 2010 and 2019, without cancer history, were included and followed up until December 2019.

**Method:**

The interaction patterns among factors associated with the risk of bladder cancer incidence were examined using conditional inference survival tree analysis.

**Results:**

A total of 382 770 patients were included. During a median follow-up of 6.2 years, 644 patients developed bladder cancer. Age (≤74 years versus >74 years) and sex emerged as primary and secondary factors in differentiating the risk of bladder cancer sequentially. Among males aged 62–74 years and males aged ≤58 years, smoking (adjusted hazard ratio [aHR] for ever versus never smoking: 1.96, 95% confidence interval [CI] = 1.49 to 2.58) and chronic kidney disease (CKD; aHR for presence versus absence: 2.92, 95% CI = 1.21 to 7.02) appeared as dominant risk factors for bladder cancer, respectively.

**Conclusion:**

This study identified the interaction patterns among age, sex, smoking, and CKD on the risk of bladder cancer incidence, providing potential targets for public health cancer prevention strategies in primary care for patients with type 2 diabetes.

## How this fits in

Previous research shows that type 2 diabetes is associated with an increased risk of bladder cancer. While older age, male sex, and smoking are known risk factors for bladder cancer, little is known about how different risk factors interact in the presence of diabetes and other lesser known factors on bladder cancer risk. Findings of the study suggest that among males aged <75 years, smoking and the presence of chronic kidney disease (CKD) appear as key risk factors in subgroups of patients. These imply the differential risk patterns across age–sex subgroups, and highlight the importance of tobacco control and renal health in bladder cancer prevention in primary care for patients with type 2 diabetes.

## Introduction

Globally, bladder cancer is the ninth most commonly diagnosed cancer.^1^ Prior research has shown that patients with type 2 diabetes have a 24% elevated risk of developing bladder cancer, when compared with those without diabetes.^2^ The major aetiological factor for bladder cancer is tobacco smoking.^3^ Other risk factors for bladder cancer include older age, male sex, and occupational exposure to carcinogens.^4^ Some evidence also suggests a potential association between chronic kidney disease (CKD) and bladder cancer.^5^

Age, male sex, and smoking are established risk factors for bladder cancer. Previous research has shown that males are four times more likely to be diagnosed with bladder cancer than females,^6^ and active smoking contributes to approximately 50% of bladder cancer risk.^7^ Also, emerging evidence suggests that CKD could be potentially linked to urinary tract cancers such as bladder cancer.^5^ However, most studies focus on patients with advanced CKD who need dialysis or renal transplant.^5^ It remains less clear whether CKD at less severe levels is also associated with bladder cancer.^5^

Nevertheless, little is known about how these known (or potential) risk factors interact in the presence of other lesser known factors to collectively influence the risk of bladder cancer. For example, active smoking itself may contribute to the development of metabolic disorders^8^ and type 2 diabetes.^9^ However, it is less clear how the co-existence of smoking exposure and diabetes may influence the course of bladder cancer development. While active smoking is an independent risk factor for bladder cancer across both sexes,^7^ there are potentially substantial differences in smoking prevalence between sexes. The relative importance of smoking on bladder cancer is less certain in comparison with other factors across different subgroups with varying smoking prevalence and cumulative exposure. Moreover, it remains largely unknown whether there are other lesser known factors that may play a key role in differentiating the risk of bladder cancer among certain subgroups. Furthermore, the presence of CKD could be associated with diabetes-associated microangiopathy; it could also be driven by other underlying causes, such as ageing,^10^ immunity, vascular system, toxicity, infection, mechanics, and genetics.^11^ In addition, despite patients with diabetes being at greater risk of developing bladder cancer, studies examining factors associated with the risk of bladder cancer or their interactions among patients with diabetes remain rare.

To identify targets for public health cancer prevention strategies, this study aims to examine the interaction patterns among factors associated with the risk of bladder cancer incidence among patients who received diabetes management in primary care using a conditional inference survival tree approach.

## Method

The method is described in Appendix 1, Supplementary materials.

## Results

**Figure 1.**
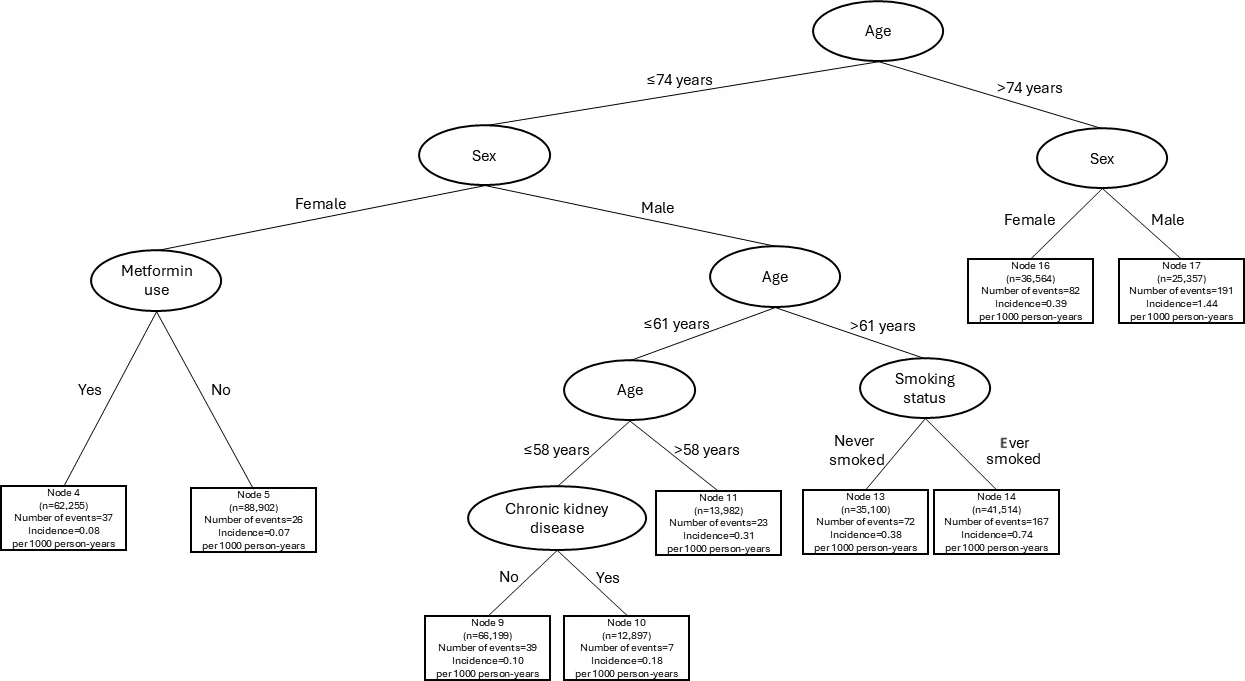
Survival tree diagram for bladder cancer incidence among patients with type 2 diabetes

Of the 382 770 patients included, 644 patients developed bladder cancer during follow-up (median 6.2 years, interquartile range [IQR] 3.3–8.0 years). The overall bladder cancer incidence was 0.30 per 1000 person–years. The incidence among females was 0.13 per 1000 person–years and among males was 0.47 per 1000 person–years. The tree model first broadly divided patients by age (≤74 years versus >74 years), followed by sex. Among males aged ≤74 years, smoking and CKD emerged as key risk factors for bladder cancer in males aged 62–74 years and those aged ≤58 years, respectively (Figure 1). Among younger males, the prevalence of smoking was 52.63% and of CKD was 16.91%, whereas for their female counterparts smoking prevalence was 6.05% and CKD prevalence was 12.07% (Table 1).

**Table 1.** Baseline characteristics of patients by age and sex

	Female, ≤74 **years**		Male, ≤74 **years**		Female, >74 **years**		Male, >74 **years**	
**Characteristics**	(***n* = 151 157**)		**(*n* = 169 692)**		**(*n* = 36 564)**		**(*n* = 25 357)**	
Number of bladder cancer cases during follow-up, *n* (%)	63	(0.04%)	308	(0.18%)	82	(0.22%)	191	(0.75%)
Demographics								
Age at assessment in years, mean±SD	59.43	±9.17	58.52	±9.24	80.39	±4.30	79.75	±4.02
Duration of diabetes in years, median (IQR)	3	(1–8)	3	(1–8)	7	(2–14)	6	(1–13)
Medical history								
Chronic kidney disease, *n* (%)	18 245	(12.07%)	28 695	(16.91%)	6195	(16.94%)	5303	(20.91%)
Cirrhosis, *n* (%)	2722	(1.80%)	3987	(2.35%)	506	(1.38%)	371	(1.46%)
Ischaemic heart disease, *n* (%)	5429	(3.59%)	14 567	(8.58%)	3963	(10.84%)	3936	(15.52%)
Cerebrovascular disease, *n* (%)	6047	(4.00%)	9941	(5.86%)	4063	(11.11%)	3639	(14.35%)
Heart failure, *n* (%)	1668	(1.10%)	2880	(1.70%)	1890	(5.17%)	1332	(5.25%)
Hypertension, *n* (%)	124 728	(82.52%)	141 511	(83.39%)	35 172	(96.19%)	24 060	(94.89%)
Chronic obstructive pulmonary disease, *n* (%)	203	(0.13%)	1280	(0.75%)	323	(0.88%)	967	(3.81%)
Pneumonia, *n* (%)	2692	(1.78%)	4786	(2.82%)	2238	(6.12%)	2275	(8.97%)
Family history of diabetes, *n* (%)	79 070	(52.31%)	85 672	(50.49%)	10 178	(27.84%)	5931	(23.39%)
Medication use								
Anti-diabetic drugs								
Metformin, *n* (%)	62 255	(41.19%)	67 027	(39.50%)	16 084	(43.99%)	9801	(38.65%)
Sulfonylurea, *n* (%)	38 260	(25.31%)	44 963	(26.50%)	12 146	(33.22%)	8206	(32.36%)
Insulin, *n* (%)	8561	(5.66%)	11 457	(6.75%)	2085	(5.70%)	1769	(6.98%)
Dipeptidyl peptidase-4 inhibitors, *n* (%)	5029	(3.33%)	7167	(4.22%)	1383	(3.78%)	1141	(4.50%)
Sodium-glucose cotransporter-2 inhibitors, *n* (%)	321	(0.21%)	662	(0.39%)	28	(0.08%)	31	(0.12%)
Glucagon-like peptide-1 receptor agonists, *n* (%)	76	(0.05%)	117	(0.07%)	2	(0.01%)	2	(0.01%)
Glucosidase inhibitors, *n* (%)	594	(0.39%)	652	(0.38%)	169	(0.46%)	136	(0.54%)
Glitazone, *n* (%)	66	(0.04%)	648	(0.38%)	95	(0.26%)	10	(0.04%)
Meglitinide, *n* (%)	594	(0.39%)	52	(0.03%)	15	(0.04%)	75	(0.30%)
Any of the above, *n* (%)	77 318	(51.15%)	89 206	(52.57%)	21 252	(58.12%)	14 013	(55.26%)
Aspirin, *n* (%)	20 761	(13.73%)	36 798	(21.69%)	12 191	(33.34%)	10 050	(39.63%)
Non-steroidal anti-inflammatory drugs, *n* (%)	90 056	(59.58%)	84 165	(49.60%)	20 170	(55.16%)	11 882	(46.86%)
Anti-coagulants, *n* (%)	4277	(2.83%)	9756	(5.75%)	2510	(6.86%)	2320	(9.15%)
Anti-platelets, *n* (%)	6282	(4.16%)	14 630	(8.62%)	3376	(9.23%)	3303	(13.03%)
Anti-hypertensive drugs, *n* (%)	98 839	(65.39%)	110 888	(65.35%)	31 687	(86.66%)	21 314	(84.06%)
Statins, *n* (%)	74 006	(48.96%)	84 071	(49.54%)	19 196	(52.50%)	13 130	(51.78%)
Behaviours								
Ever drinking, *n* (%)	17 812	(11.78%)	80 882	(47.66%)	2710	(7.41%)	9520	(37.54%)
Ever smoking, *n* (%)	9139	(6.05%)	89 304	(52.63%)	2472	(6.76%)	13 885	(54.76%)
Anthropometric measurements								
Body mass index in kg/m^2^, mean±SD	26.26	±4.45	26.29	±4.07	25.16	±3.83	24.92	±3.30
Waist-to-hip ratio, mean±SD	0.92	±0.06	0.95	±0.06	0.94	±0.07	0.96	±0.06
Laboratory measurements								
HbA1c in %, mean±SD	7.36	±1.39	7.46	±1.59	7.07	±1.12	7.12	±1.24
Fasting glucose in mmol/l, mean±SD	7.65	±2.24	7.75	±2.38	7.22	±1.89	7.22	±1.90
Low-density lipoprotein cholesterol in mmol/l, mean±SD	2.77	±0.85	2.64	±0.81	2.61	±0.82	2.47	±0.76
High-density lipoprotein cholesterol in mmol/l, mean±SD	1.35	±0.34	1.18	±0.30	1.37	±0.36	1.23	±0.34
Triglycerides in mmol/l, mean±SD	1.62	±1.09	1.67	±1.35	1.53	±0.84	1.34	±0.81
Serum creatinine in µmol/l, mean±SD	67.69	±31.46	89.89	±45.65	84.51	±36.70	106.99	±44.74

HbA1c = glycated haemoglobin

### Age and sex

**Figure 2.**
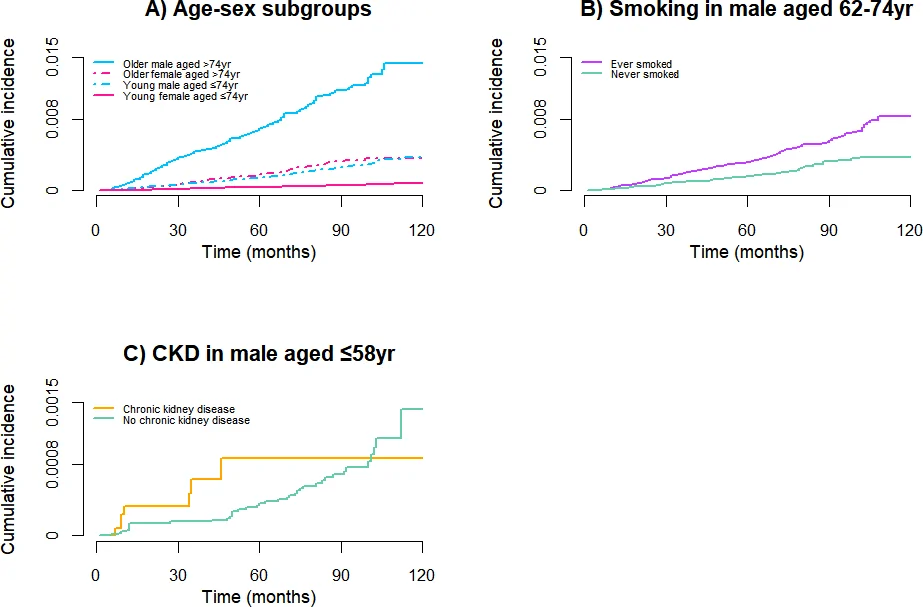
Cumulative bladder cancer incidence (A) across age–sex subgroups; (**B**) across those who had ever smoked and never smoked among males aged 62–74 years; (**C**) across males aged ≤58 years in the presence or absence of chronic kidney disease. CKD = chronic kidney disease

The tree model first identified age at 74 years as a primary factor in differentiating the risk of bladder cancer within the study diabetes cohort. Among patients aged >74 years, males tended to have a higher risk of developing bladder cancer than females (adjusted hazard ratio [aHR] 3.89, 95% CI = 3.00 to 5.04), adjusting for age and duration of diabetes (Table 2). Younger males were further partitioned into several age groups characterised by different dominant risk factors for bladder cancer (Figure 1). On the other hand, females aged ≤74 years generally had the lowest risk of bladder cancer regardless of metformin use (Figures 1 and 2A;Table 2).

**Table 2.** Comparisons in adjusted hazard ratios of selected split variables between sibling nodes

Node	Characteristics	Comparison i, Metformin use in females aged ≤74 years	Comparison ii, Chronic kidney disease in males aged ≤58 years	Comparison iii, Smoking in males aged 62–74 years	Comparison iv, Male sex in those aged >74 years
Node 4	{Female, aged ≤74 years, metformin use}	1			
Node 5	{Female, aged ≤74 years, no metformin use}	1.13 (0.67 to 1.91)			
Node 9	{Male, aged ≤58 years, no chronic kidney disease}		1		
Node 10	{Male, aged ≤58 years, chronic kidney disease}		2.92 (1.21 to 7.02)*		
Node 13	{Male, aged between 62 years and 74 years, never smoked}			1	
Node 14	{Male, aged between 62 years and 74 years, has ever smoked}			1.96 (1.49 to 2.58)*	
Node 16	{Female, aged >74 years}				1
Node 17	{Male, aged >74 years}				3.89 (3.00 to 5.04)*

Note 1: comparisons were made with adjustment of age and duration of diabetes. Note 2: adjusted hazard ratios (aHRs) are presented in 95% confidence interval (CI). Note 3: statistical significance was set at an alpha level of 0.05.

**Bold** and asterisked(*) = statistically significant.

### Age, male sex, and smoking

Among males aged 62–74 years, smoking was identified as a dominant risk factor for bladder cancer (Figure 1). Those who had ever smoked had an increased risk of developing bladder cancer when compared with those who had never smoked (aHR 1.96, 95% CI = 1.49 to 2.58), controlling for age and duration of diabetes (Table 2;Figure 2B).

### Age, male sex, and chronic kidney disease

Among males aged ≤58 years, CKD appeared as a key risk factor for bladder cancer (Figure 1). Those with CKD had an elevated risk of developing bladder cancer when compared with those without CKD (aHR 2.92, 95% CI = 1.21 to 7.02), adjusting for age and duration of diabetes (Table 2). Nevertheless, those with CKD tended to have a shorter follow-up when compared with those without CKD (median: 3.1 versus 6.9 years). Those with CKD reached a plateau level of cumulative bladder cancer incidence at 3.9 years. The cumulative incidence for those without CKD reached the same level 4.5 years later (at 8.4 years) (Figure 2C).

### Model performance

The area under the curves (AUCs) at 2 years, 5 years, and 7 years were 0.813 (95% CI = 0.813 to 0.813), 0.778 (95% CI = 0.777 to 0.779), and 0.782 (95% CI = 0.781 to 0.783), respectively.

## Discussion

### Summary

The current study showed that despite older age, male sex, and smoking being established risk factors for bladder cancer, these factors may play differential roles in influencing the risk of bladder cancer among subgroups of patients who received diabetes management in primary care. The survival tree model broadly partitioned patients into several subgroups to differentiate their risk of bladder cancer according to age and sex, and identified predominant factors associated with bladder cancer risk within age–sex subgroups. Findings of the study suggested a notable increased risk of bladder cancer among patients who had reached 75 years at baseline. In addition, among younger males aged ≤74 years, smoking exposure, and the presence of CKD appeared as important risk factor for bladder cancer for those aged 62–74 years and aged ≤58 years, respectively.

### Comparison with existing literature

In the present study, smoking emerged as a dominant factor in determining the risk of bladder cancer among males aged 62–74 years with diabetes. Prior research has shown that active smoking itself is associated with an increased risk of metabolic disorders^8^ and type 2 diabetes.^9^ One possible mechanism linking smoking to metabolic abnormalities is that nicotine exposure may impair insulin sensitivity and promote insulin resistance.^12,13^ On the other hand, smoking is also a known risk factor for bladder cancer.^3^ Possible mechanisms include direct exposure to carcinogens in tobacco and indirect exposure to a chronic low-grade inflammatory environment conducive to carcinogenesis.^14,15^

The epidemiological association between smoking and bladder cancer has been observed across both sexes.^7^ Nevertheless, in the present study cohort, there exists a large gap in smoking prevalence between two sexes (male: 53% to 55% versus female: 6% to 7%). The relatively low prevalence of smoking among females in the study cohort may partially contribute to smoking being a less important factor in differentiating the risk of bladder cancer among females. In addition, a recent review^16^ has summarised that there are potential biological differences in carcinogenesis of the bladder between two sexes, potentially accounting for the differential incidence rates and outcomes between sexes. Some potential key factors include differences in anatomical, immunological, sex hormonal, epigenetic, and genetic characteristics.^16^

Furthermore, the emergence of smoking as a dominant factor in differentiating the risk of bladder cancer among males aged 62–74 years could be owing to cumulative exposure over the life course. A previous meta-analysis^17^ has quantified the dose-response association between tobacco smoking exposure and bladder cancer risk, demonstrating the gradual increased risk with smoking exposure up to 50 pack–years and a risk plateau afterwards, as well as a general increasing trend with smoking duration up to 60 years. Findings of the study are generally consistent with the effects of cumulative exposure to smoking on bladder cancer risk found in the literature.^17^

Moreover, CKD emerged as a key factor in differentiating the risk of bladder cancer among males aged ≤58 years with diabetes. Those with CKD generally had a higher risk of developing bladder cancer up to 8.4 years when compared with those without CKD. The plateau reached by those with CKD early at 3.9 years could be in part owing to small number of bladder cancer cases (*n* = 7), and lower life expectancy resulting in shorter follow-up. In the literature, emerging evidence suggests that the presence of CKD could be potentially associated with an elevated risk of bladder cancer.^5^ Nevertheless, whether renal dysfunction at a less severe stage is associated with bladder cancer risk remains controversial.^5,18,19^ For example, Lowrance *et al* found that only patients with estimated glomerular filtration rate (eGFR) <30 ml/min per 1.73 m^2^ demonstrated an increased risk of bladder cancer.^18^ On the other hand, a meta-analysis summarised that reduced renal function is only associated with urinary tract cancers among patients on dialysis.^19^ In addition, the kidney is a target organ of microangiopathy under diabetes condition.^20^ The presence of CKD could be a complication of diabetes resulting from progression of diabetes, potentially indicating a more advanced stage over the course of diabetes progression. Given diabetes is a common risk factor for both CKD and cancer,^5^ the presence of CKD in diabetes may accelerate carcinogenesis of bladder cancer.

A previous study^21^ showed that the coexistence of CKD and diabetes may exert additive positive effects on the risk of cancer mortality. Potential pathophysiological mechanisms linking CKD and bladder cancer include underlying cystic kidney disease, accumulation of toxins as a consequence of renal dysfunction via different underlying causes, chronic inflammation, and oxidative stress.^5^ Moreover, CKD is an age-related condition.^22^ Previous research suggests that a healthy 75-year-old individual only has half the nephrons they had at 25 years old.^11^ It is also suggested that patients with CKD are physiologically 5 years older than their actual age.^10^ The presence of CKD may imply premature ageing^22^ among younger individuals. Furthermore, the unique emergence of CKD as a key factor among males but not females among the study cohort could be owing to differences between sexes in CKD prevalence, life expectancy, declining rates of renal functioning, sex hormones, and risk preferences for CKD treatment.^23^ Possible mechanisms linking sex differences to CKD include direct effects of sex hormones, differences in nitric oxide metabolism and oxidative stress in the kidneys, potentially owing to interactions with sex hormones, as well as differential lifestyle factors between sexes.^23^

Overall, findings of the current study add to the literature by identifying the potential interaction patterns among age, sex, smoking, and CKD on bladder cancer risk among patients who received routine diabetes management in primary care. The emergence of age at 75 years as the identified optimal cutoff for elevated bladder cancer risk coincides with the expected half-life of total renal mass during natural ageing. While smoking and renal dysfunction is a known risk factor or potential risk factor for bladder cancer, in the presence of diabetes, smoking and CKD emerged as key risk factors for males aged 62–74 years and ≤58 years, respectively. Active smoking itself may contribute to the onset of type 2 diabetes,^9^ and the concurrent presence of smoking exposure and diabetes later in life may collectively influence the course of bladder cancer development. On the other hand, the presence of CKD is often considered as a sign of accelerated ageing,^22^ or underlying comorbid conditions in diabetes such as microvascular and macrovascular complications.^20^ The premature ageing of kidneys in diabetes, likely in the presence of multiple comorbidities, may ultimately aggravate carcinogenesis of the bladder along the urinary tract. Furthermore, the emergence of smoking and CKD as important risk factors in younger males aged <75 years may attribute to the differences in smoking prevalence across sexes in the study cohort, and differential CKD patterns across sexes.^23^

### Implications for research and practice

There are several public health implications of the present study. First, age–sex subgroups with differential risk profiles for bladder cancer among the diabetes cohort were identified. For patients with type 2 diabetes in the absence of bladder cancer symptoms, older males, aged 62–74 years males who had ever smoked, and youngest males with CKD, could be at higher risk of developing bladder cancer. Closer monitoring of potential bladder cancer symptoms during routine follow-up for diabetes management in primary care may help identify bladder cancer cases early.

Second, while smoking is a well-established risk factor for bladder cancer, smoking is a particularly dominant risk factor among males aged 62–74 years with diabetes. Smoking prevention in primary care targeted at the broader general population, in particular before the onset of type 2 diabetes, may help reduce the risk of bladder cancer.

Third, the presence of CKD could be potentially associated with an elevated risk of bladder cancer and act as a key risk factor among males aged ≤58 years with diabetes. Preventing renal dysfunction through regular monitoring and potential dietary intervention^10^ (for example, protein restriction^10^ and plant-based diet)^24^ during routine diabetes management in primary care may help lower the risk of developing bladder cancer. Specifically, management of blood pressure, glucose, and lipids, as well as lifestyle modifications may potentially help reduce the risk of developing diabetes-associated kidney disease^20^ Furthermore, prior research suggests that patients with CKD are biologically older than their chronological age.^10^ The presence of CKD may indicate a suboptimal general health status. Specifically, the presence of CKD in diabetes is considered as a marker of underlying comorbid conditions, such as other diabetes-associated microvascular and macrovascular complications.^20^

Fourth, the emergence of several dominant risk factors among males may highlight differences in epidemiology and support the potential differential underlying mechanisms between two sexes. Different sex-specific bladder cancer prevention strategies may help tailor to the differential needs of the two sexes.

Fifth, compared with the general population, it is unclear among the study’s cohort of people with diabetes to what extent the onset of type 2 diabetes is attributable to active smoking, and to the coexistence of smoking and other unhealthy behaviours^25^ that may collectively promote type 2 diabetes. It is also uncertain to what extent the presence of renal dysfunction is associated with diabetes alone, other underlying causes,^11^ or a combination of factors. Future research is warranted to compare findings with patients without diabetes in the general population.

In conclusion, the current study demonstrated the interaction patterns among age, sex, smoking, and CKD on the risk of bladder cancer incidence among patients who received routine diabetes care at general outpatient clinics (GOPCs). Consistent with the findings in the general population, the risk of bladder cancer generally increases with age. Males are more predisposed to bladder cancer development than females. Among the study diabetes cohort, there is a marked elevated risk once patients reach 75 years. While smoking and CKD are known or potential risk factors for bladder cancer among the general population, in the study cohort, these two factors emerge as important risk factors in particular to males aged 62–74 years and males ≤58 years, respectively. Findings of the study may provide potential targets for public health cancer prevention strategies in primary care for patients with type 2 diabetes.

### Strengths and limitations

Strengths of the study include a large cohort with regular follow-up in GOPCs; relative comprehensive assessment at baseline to capture the clinical profile of individual patients with type 2 diabetes; the homogenous profile of patients who receive diabetes care in GOPCs; and the linkage across datasets in the electronic health records system to capture clinical information of patients. However, several limitations may exist in the current study. First, information on some potential confounders such as dosage of smoking exposure (age of smoking initiation or pack–years of smoking), occupation, and dietary exposure was not available. Second, severity of CKD was not differentiated. Patients with more advanced stages of CKD may not survive long enough to experience the event of bladder cancer diagnosis. Nevertheless, patients with CKD appeared to have a higher risk of developing bladder cancer up to 8.4 years of follow-up. Further studies are needed to examine at which stage of CKD the risk of bladder cancer may potentially increase. Third, the underlying causes of CKD within the diabetes cohort were not distinguishable. Lastly, given type 2 diabetes as an age-related chronic disease,^26^ the role of smoking in metabolic disorders^8^ and type 2 diabetes,^9^ as well as diabetes-associated kidney disease as a renal manifestation of microangiopathy under diabetes condition,^20^ future research is warranted to examine generalisability of the findings to other diabetes populations and the broader general population. In addition, the optimality of the identified cutoffs for age among the study diabetes population may also need further verification in other populations.

## Data

Data is not available for sharing due to access restriction.
